# Bacterial pathogenesis of plants: future challenges from a microbial perspective: Challenges in Bacterial Molecular Plant Pathology

**DOI:** 10.1111/mpp.12427

**Published:** 2016-08-04

**Authors:** Sebastian Pfeilmeier, Delphine L. Caly, Jacob G. Malone

**Affiliations:** ^1^ The Sainsbury Laboratory, Norwich Research Park Norwich NR4 7UH UK; ^2^ John Innes Centre, Norwich Research Park Norwich NR4 7UH UK; ^3^ Université de Lille, EA 7394, ICV ‐ Institut Charles Viollette Lille F‐59000 France; ^4^ University of East Anglia Norwich NR4 7TJ UK

**Keywords:** bacterial signalling, phytotoxins, plant infection, plant pathogenicity, *Pseudomonas*, type III effectors, *Xanthomonas*

## Abstract

**Future Challenges in Plant Pathology**

Plant infection is a complicated process. On encountering a plant, pathogenic microorganisms must first adapt to life on the epiphytic surface, and survive long enough to initiate an infection. Responsiveness to the environment is critical throughout infection, with intracellular and community‐level signal transduction pathways integrating environmental signals and triggering appropriate responses in the bacterial population. Ultimately, phytopathogens must migrate from the epiphytic surface into the plant tissue using motility and chemotaxis pathways. This migration is coupled with overcoming the physical and chemical barriers to entry into the plant apoplast. Once inside the plant, bacteria use an array of secretion systems to release phytotoxins and protein effectors that fulfil diverse pathogenic functions (Fig. 1) (Melotto and Kunkel, 2013; Phan Tran *et al*., 2011).

As our understanding of the pathways and mechanisms underpinning plant pathogenicity increases, a number of central research challenges are emerging that will profoundly shape the direction of research in the future.

- We need to understand the bacterial phenotypes that promote epiphytic survival and surface adaptation in pathogenic bacteria. How do these pathways function in the context of the plant‐associated microbiome, and what impact does this complex microbial community have on the onset and severity of plant infections?
- The huge importance of bacterial signal transduction to every stage of plant infection is becoming increasingly clear. However, there is a great deal to learn about how these signalling pathways function in phytopathogenic bacteria, and the contribution they make to various aspects of plant pathogenicity.
- We are increasingly able to explore the structural and functional diversity of small‐molecule natural products from plant pathogens. We need to acquire a much better understanding of the production, deployment, functional redundancy and physiological roles of these molecules.

Type III secretion systems (T3SSs) are important and well‐studied contributors to bacterial disease. Several key unanswered questions will shape future investigations of these systems.

- We need to define the mechanism of hierarchical and temporal control of effector secretion.
- For successful infection, effectors need to interact with host components to exert their function. Advanced biochemical, proteomic and cell biological techniques will enable us to study the function of effectors inside the host cell in more detail and on a broader scale.
- Population genomics analyses provide insight into evolutionary adaptation processes of phytopathogens. The determination of the diversity and distribution of type III effectors (T3Es) and other virulence genes within and across pathogenic species, pathovars and strains will allow us to understand how pathogens adapt to specific hosts, the evolutionary pathways available to them, and the possible future directions of the evolutionary arms race between effectors and molecular plant targets.

Although pathogenic bacteria employ a host of different virulence and proliferation strategies, as a result of the space constraints, this review focuses mainly on the hemibiotrophic pathogens. We discuss the process of plant infection from the perspective of these important phytopathogens, and highlight new approaches to address the outstanding challenges in this important and fast‐moving field.

**Figure 1:**
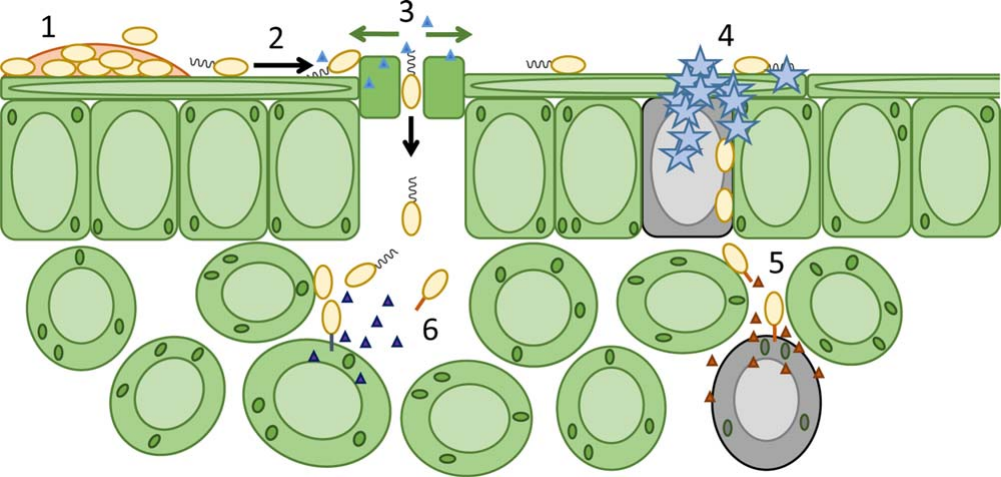
The process of plant infection. 1, Surface survival and biofilm formation. 2, Flagella/pili‐driven migration across plant surfaces to apoplastic entry sites. 3, Release of phytotoxins to bypass stomatal closure. 4, Ice nucleation to damage plant surfaces. 5, Extracellular enzyme secretion to degrade cell walls and damage plant tissue. 6, Phytotoxin secretion to modify plant physiology, metabolism and immune responses.

## Introduction—Surviving Stress on the Plant Epiphytic Surface

The surface of plant leaves is a harsh environment for pathogenic microorganisms, with bacteria regularly exposed to desiccation, ultraviolet irradiation, adverse temperature changes and mechanical disruption, for example, by strong winds. Despite these challenges, epiphytic bacteria often reach population densities of between 10^6^ and 10^7^ cells/cm^2^ of leaf (Andrews and Harris, 2000). Although indirect, microbial persistence on the plant surface may be considered as a virulence strategy, as plant disease symptoms are effectively reduced by the disruption of pathways involved in epiphytic survival. Furthermore, surface survival pathways may also play important roles during other stages of infection.

Metabolic responses to cold and osmotic shock, and desiccation, stress materially contribute to epiphytic survival (Djonovic *et al*., 2013; Freeman *et al*., 2013; Wu *et al*., 2012). For example, the osmoprotectant trehalose has been implicated in *Pseudomonas syringae* survival and population maintenance in the phyllosphere (Freeman *et al*., 2010), and has also been linked to plant pathogenicity in the opportunist *Pseudomonas aeruginosa*, where it has been suggested to promote the acquisition of nitrogen, enhancing proliferation in the leaf apoplast (Djonovic *et al*., 2013). Exopolysaccharide (EPS) production plays an important role in the epiphytic survival of the plant‐associated microbiome, including pathogenic *Xanthomonas* spp. (Dunger *et al*., 2007) and *P. syringae* (Yu *et al*., 1999). Different EPS molecules have been shown to contribute to plant surface colonization and infection, including the well‐studied polymers xanthan (Dunger *et al*., 2007), levan and alginate (Freeman *et al*., 2013; Laue *et al*., 2006; Yu *et al*., 2013), although a large number of novel EPSs undoubtedly exist in nature whose role in plant pathogenicity remains to be determined. For example, the acetylated cellulose Wss is important for effective root colonization by the commensal bacterium *Pseudomonas fluorescens* (Gal *et al*., 2003), and is also present in *P. syringae* pv. *tomato* (*Pto*) DC3000 (Prada‐Ramirez *et al*., 2016). The aggressive kiwi pathogen *P. syringae* pv. *actinidiae* (*Psa*) NZ V‐13 produces two novel, previously uncharacterized, polysaccharides: a branched α‐d‐rhamnan and an α‐d‐1,4‐linked glucan polymer (Ghods *et al*., 2015). The genomes of *P. syringae* pathovars also encode a number of EPS loci that have been characterized in other *Pseudomonas* species, but whose contribution to plant infection is undefined. These include the widespread *pel* and *psl* operons (Winsor *et al*., 2011), which play important roles in persistence during cystic fibrosis lung infections by the opportunistic pathogen *P. aeruginosa* (Malone, 2015).

EPS molecules are associated with a number of different plant interaction functions, most of which are linked to epiphytic survival, contributing to freeze–thaw resistance (Wu *et al*., 2012), desiccation and osmotic stress tolerance (Chang *et al*., 2007; Freeman *et al*., 2013), and maintenance of the microbial population (Dunger *et al*., 2007). In addition to their roles in protecting bacteria on the plant surface, EPS molecules from several phytopathogens have also been implicated in immune evasion, possibly mediated by the quenching of calcium signalling during the plant immune response (Aslam *et al*., 2008), whereas the levan biosynthetic locus in *P. syringae* pv. *syringae* (*Pss*) B728a is strongly up‐regulated in the apoplast relative to the leaf surface, suggesting a further, post‐infection role in virulence (Yu *et al*., 2013). Laue *et al*. (2006) have suggested that accumulated levan may function as a nutrient storage source to enable later stages of biofilm formation, although it remains to be determined whether levan fulfils a similar role during plant infections. Finally, *Psa* NZ V‐13 forms biofilms on both external and internal plant surfaces at different stages of infection, again consistent with an apoplastic role for phytopathogenic EPS (Renzi *et al*., 2012).

## Signal Transduction in Plant Pathogens

Life on plant leaves is both complex and dynamic, with epiphytic pathogens continually deciding whether to express traits that enable them to survive on the surface, or to bypass the leaf epidermis and enter the apoplast, before propagating in the intercellular space or colonizing the plant vascular system. This decision‐making process is a critical factor in the onset, virulence and persistence of plant infections, and is mediated, to a large degree, by small‐molecule‐based signalling pathways. These systems regulate motility and biofilm formation, the expression of virulence genes and communication with other bacteria, both within and between species. In plant pathogens, a large number of signalling systems have been described, with the main ones being quorum sensing (QS) and second messenger pathways, such as cyclic guanosine 3',5'‐monophosphate (cdG) signalling. Here, we focus our attention on signalling in *Xanthomonas* spp. and *P. syringae*. These economically important, model phytopathogens use different, well‐characterized signalling systems: *Xanthomonas campestris* pv. *campestris* (*Xcc*) produces a fatty acid QS signal called diffusible signal factor (DSF), which is linked to cdG signalling. QS in *Pss*, however, is mediated by the acyl‐homoserine lactones (AHLs), a widely distributed QS signal among Gram‐negative bacteria. Although there is a body of work on cdG signalling in *Xanthomonas* (see below), the role of cdG signalling in *P. syringae* pathogenesis has only been described recently (Engl *et al*., 2014; Perez‐Mendoza *et al*., 2014; Pfeilmeier *et al*., 2016).

## QS in Plant Pathogens

**Figure 2:**
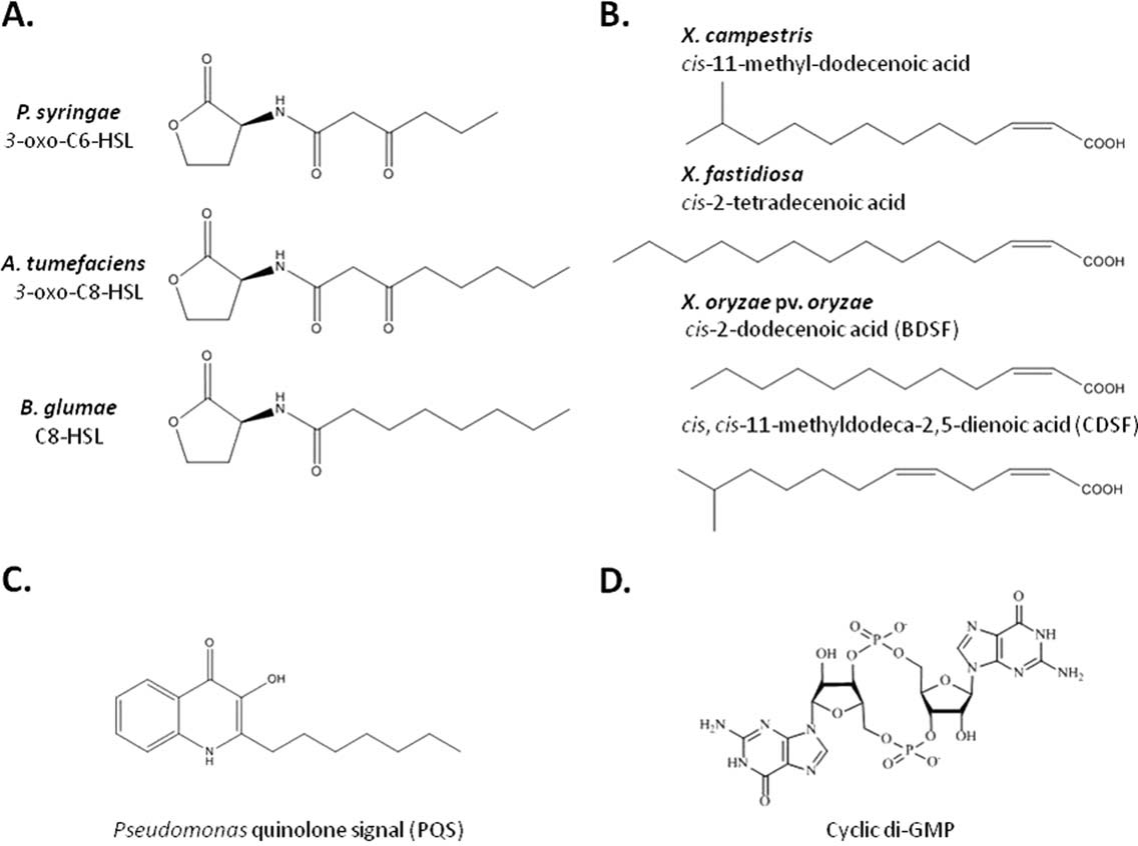
Structural diversity of signalling molecules in plant pathogens. (A) *N*‐Acyl homoserine lactones (HSL, homoserine lactone). (B) Fatty acid diffusible signal factor (DSF)‐like signals. (C) *Pseudomonas* quinolone signal. (D) The second messenger cyclic di‐guanosine monophosphate (cyclic di‐GMP).

Bacteria use QS to communicate and assess cell density by producing small signalling molecules, called auto‐inducers, which are sensed by bacteria in the immediate environment and affect gene expression once a threshold level reflecting a critical cell density is attained. A large number of QS molecules have been described, with various roles and high structural diversity (Fig. 2). In plant‐pathogenic bacteria, the major characterized signals are AHLs, DSF and Ax21, which are discussed in more detail below.

AHLs represent a major class of QS molecules in plant pathogens. Most AHL systems consist of two genes, coding for an AHL receptor and an AHL synthase. Transcription of the synthase gene is positively regulated by the AHL itself. *Pseudomonas syringae* produces the AHL molecule 3‐oxo‐C6‐HSL (HSL, homoserine lactone) (Quinones *et al*., 2004). The *ahlI*/*ahlR* system in *Pss* regulates motility and EPS production, essential factors for plant colonization and virulence (Quinones *et al*., 2005). The crown gall pathogen *Agrobacterium tumefaciens* produces 3‐oxo‐C8‐HSL to control virulence through the stimulation of Ti plasmid copy numbers, whereas, in the rice pathogen *Burkholderia glumae*, the production of C8‐HSL regulates phytotoxin secretion and motility through control of flagellum assembly (Kim *et al*., 2004; Pappas and Winans, 2003). Some bacteria produce several AHLs. This is the case for *P. aeruginosa*, which uses three AHL pathways (LasI/LasR, RhlI‐RhlR and the orphan regulator QscR) to produce 3‐oxo‐C12‐HSL and C4‐HSL, which affect the expression of more than 300 genes, many involved in toxin secretion, motility and biofilm formation (Schuster *et al*., 2003). *Pseudomonas aeruginosa* also produces a third QS molecule, 2‐heptyl‐3‐hydroxyl‐4‐quinolone (also called PQS for *Pseudomonas* quinolone signal), involved in the production of virulence factors and the control of biofilm formation (Allesen‐Holm *et al*., 2006; Pesci *et al*., 1999). The expression of *lasB*, which encodes a type II secreted protease, has been shown to be mediated by the *las*, *rhl* and PQS systems in *P. aeruginosa*, whereas PQS synthesis is also controlled by the two AHL signals, highlighting the complexity of gene regulation through QS signalling systems (Pesci *et al*., 1999). Thus far, PQS‐like signals have not been detected in other phytopathogenic pseudomonads.

Another QS signal of interest is the recently described protein Ax21. Ax21 is a small sulfated protein produced by the rice pathogen *Xanthomonas oryzae* pv. *oryzae*, which acts as a QS molecule required for virulence gene expression (Han *et al*., 2011). Similar proteins have subsequently been identified in other pathogens, including *X. oryzae* pv. *oryzicola* (Qian *et al*., 2013) and the commensal *Stenotrophomonas maltophilia* (McCarthy *et al*., 2011), and have been shown to be essential for virulence, biofilm formation, motility and EPS production. However, the Ax21 homologue in *Xylella fastidiosa* was shown not to be required for virulence or biofilm formation, supporting the species‐specific evolution of QS signal‐mediated regulation of virulence (Pierce *et al*., 2014).

*Xanthomonas* species use DSF, which is a different class of QS molecule composed of unsaturated fatty acids. The DSF signal sensed by *Xcc*, a pathovar that causes black rot in crucifers, is *cis*‐11‐methyl‐dodecenoic acid (Wang *et al*., 2004) (Fig. 2) and is required for virulence and to regulate xanthan and protease synthesis (Barber *et al*., 1997; Tang *et al*., 1991). Since its original description, the DSF family has expanded, with new molecules having a similar unsaturated fatty acid backbone, but showing some variation in structure. For example, *Xy. fastidiosa* DSF is *cis*‐2‐tetradecenoic acid (Beaulieu *et al*., 2013). Depending on the growth conditions, *X. oryzae* pv. *oryzae* produces DSF and two other molecules: *cis*‐2‐dodecenoic acid (similar to the signal produced by *Burkholderia cenocepacia*, BDSF) and *cis*,*cis*‐11‐methyldodeca‐2,5‐dienoic acid (CDSF) (Fig. 2) (He *et al*., 2010).

**Figure 3:**
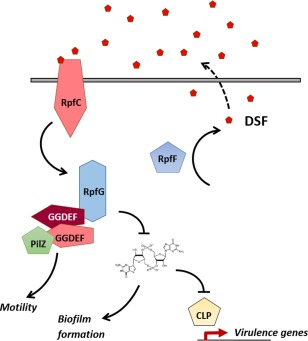
The Rpf (regulation of pathogenicity factor) hybrid quorum sensing/cyclic di‐guanosine monophosphate (QS/cdG) signalling system in *Xanthomonas* spp. On sensing the diffusible QS signal diffusible signal factor (DSF) (produced by the synthase RpfF), the sensor kinase RpfC phosphorylates and activates the HD‐GYP phosphodiesterase (PDE) RpfG. The PDE activity of RpfG degrades cdG, leading to a reduction in biofilm formation and release of Clp (for Crp‐like protein) repression. This, in turn, leads to Clp‐mediated virulence gene transcription. RpfG also interacts with several GGDEF domain‐containing proteins, which, in turn, regulate bacterial motility.

DSF signalling is mediated by products of the *rpf* (*regulation of pathogenicity factor*) gene cluster in *Xcc*, the mutation of which leads to reduced virulence (Barber *et al*., 1997). DSF is synthesized by the putative enoyl‐CoA hydratase RpfF, whereas the fatty acyl‐CoA ligase RpfB has been implicated in signal turnover (Barber *et al*., 1997; Bi *et al*., 2014; Zhou *et al*., 2015). It is then sensed by RpfC, a hybrid sensor kinase, which also negatively regulates DSF synthesis (Slater *et al*., 2000). RpfC is part of a two‐component system with the response regulator RpfG. RpfG consists of a phosphorylatable receiver domain and an HD‐GYP domain, with phosphodiesterase (PDE) activity involved in cdG degradation (Slater *et al*., 2000). On signal transduction through the RpfC/RpfG system, the expression of virulence genes is activated by cdG degradation and release of Clp (for Crp‐like protein) transcription regulator repression (Fig. 3) (Chin *et al*., 2010; Ryan *et al*., 2006). In addition to RpfC, a further DSF sensor, RpfS, has been described recently in *Xcc* and has been shown to be required for virulence to Chinese radish (An *et al*., 2014a). It is thus very likely that other DSF sensors still remain to be discovered.

Similar systems have been described in other plant pathogens, such as *Xy. fastidiosa* and additional *Xanthomonas* species, with some notable differences in regulatory outcomes. For example, in *Xy. fastidiosa*, *rpfF* mutation leads to increased virulence and increased colonization of grape xylem vessels (Chatterjee *et al*., 2008b; Newman *et al*., 2004). Moreover, a recent study of *Xy. fastidiosa* RpfF has shown that this DSF synthesis protein is also essential for signal transduction (Ionescu *et al*., 2013). *Xylella fastidiosa* apparently uses DSF signalling in a multifaceted manner to regulate its virulence, adhesion and colonization potential, and depending on whether it is infecting a plant or an insect (Chatterjee *et al*., 2008a, 2008a).

Although the *rpf* gene cluster appears to be conserved across *X. campestris* pathovars, differences exist between the model species *Xcc* and other species within the *Xanthomonas* genus. Depending on the species, the pathovar or even the strain, different phenotypes can be observed when mutating genes in the *rpf* cluster. For example, in *X. oryzae* pv. *oryzae*, RpfC has been shown to be required for virulence, but *rpfC* mutation has no effect on protease production (Tang *et al*., 1996). These differences are probably a result of the use of different cdG binding targets for gene regulation (see below) and further highlight the remarkable adaptive potential of plant pathogens.

## cdG Signalling

Second messenger nucleotides, such as cyclic adenosine monophosphate (cAMP) and cdG, are known to function as signalling molecules and have been shown to play numerous roles in the control of bacterial virulence. We limit our discussion of this complex field to the role of cdG in plant pathogenesis. As discussed above, QS and DSF signalling are closely linked to cdG signalling in plant pathogens and constitute additional layers of virulence regulation. cdG is a second messenger found in most bacteria and is involved in the regulation of a wide range of functions, including virulence, biofilm formation and motility. cdG was initially described in *Acetobacter xylinum* as a regulator of cellulose synthesis (Ross *et al*., 1987). Since its discovery, there has been a great deal of research into the synthesis of cdG, the proteins involved in signal transduction and its binding targets [for a detailed recent review on cdG, see Romling *et al*. (2013)]. Intracellular cdG levels are controlled by diguanylate cyclases (DGCs) and PDEs. DGCs, containing a GGDEF domain, synthesize cdG from two guanosine triphosphate (GTP) molecules, and PDEs, with an EAL or an HD‐GYP domain, degrade the second messenger. The activity of these proteins is controlled by environmental cues, thus enabling bacteria to modulate their cdG levels to respond to the environment. For this reason, DGC/PDEs often contain additional domains involved in signalling (GAF, PAS or REC domains, for example). In general, high levels of cdG promote a sessile lifestyle and biofilm formation and inhibit virulence and motility. The second messenger exerts its action by interacting with a diverse array of binding domains, including proteins with a PilZ domain, enzymatically inactive DGCs and PDEs, RNA riboswitches and transcriptional regulators, such as Clp and the *Pseudomonas* motility/EPS regulator FleQ (Romling *et al*., 2013).

In the DSF/*rpf* system, cdG interacts with Clp, preventing Clp promoter binding and thus the transcription of its target genes (Fig. 3). In *Xcc*, Clp has been shown to control the expression of genes encoding extracellular enzymes (Chin *et al*., 2010). The same interaction between Clp and cdG has been observed in *Xanthomonas axonopodis* pv. *citri* (Leduc and Roberts, 2009). Another two‐component system, RavS/RavR, required for virulence and linked to cdG, has been found in *Xcc*. The RavR protein has been shown to alter cdG levels through the PDE activity of its EAL domain and to regulate virulence factor expression via the transcriptional regulator Clp (He *et al*., 2009). The signal in this case is not DSF, but the sensing of hypoxia through the PAS domains of RavS (He *et al*., 2009). This signalling system appears to work synergistically with RpfC/RpfG and DSF signalling to ensure survival and pathogenicity in plant xylems, where low oxygen conditions are found.

The HD‐GYP PDE domain contains two conserved motifs separated by a small, less conserved region: the HD residues, which are required for enzymatic activity, and the GYP residues, which are involved in protein–protein interactions with GGDEF domains (Ryan *et al*., 2006, 2010). Mutation of either residue of the RpfG HD motif results in reduced extracellular enzyme production, and alanine substitutions of the GYP residues lead to reduced motility (Ryan *et al*., 2006, 2010). With both motifs playing a role in the regulation of virulence in *Xcc*, RpfG exerts its regulatory action through the mediation of cdG levels, but also by direct interaction with other proteins involved in the modulation of cdG levels (Fig. 3).

The role of cdG signalling in plant‐pathogenic *Pseudomonas* has only recently been addressed. cdG‐regulated systems control multiple important aspects of phytopathogenic *Pseudomonas* behaviour, and affect the virulence of various species and pathovars (Engl *et al*., 2014; Perez‐Mendoza *et al*., 2014; Pfeilmeier *et al*., 2016). A specific role of cdG has been suggested in the regulation of flagellum and T3SS activity (Trampari *et al*., 2015), and the control of proteome composition through ribosomal modification (Little *et al*., 2016). The Gac‐Rsm network, which controls secretion systems, biofilm formation and QS, as well as siderophore and toxin production (Vakulskas *et al*., 2015), affects cdG levels by manipulating PDE/DGC production (Moscoso *et al*., 2011, 2014). GacA has also been linked to T6SS expression in *P. syringae* (Records and Gross, 2010), whereas *in trans* overexpression of the RNA‐binding post‐transcriptional regulator RsmA represses the production of numerous secreted virulence factors (Kong *et al*., 2012).cdG signalling in plant pathogenesis has been mainly studied in certain model *Xanthomonas* and *Pseudomonas* species, but its regulatory roles in other important phytopathogens, such as *Ralstonia solanacearum*, are poorly understood (Genin and Denny, 2012). Members of the genera *Serratia* and *Erwinia* have also been shown to use cdG signalling during plant infection (Romling *et al*., 2013), although, once again, the fine details of the signalling networks in each case remain poorly defined. The complex environments of the soil and the phyllosphere seem to promote correspondingly complex regulatory networks to adapt and respond to environmental signals. *Xcc* contains over 30 genes coding for DGCs and PDEs (Ryan *et al*., 2006), and this species is by no means unusual among the phytopathogens. Moreover, the list of experimentally verified cdG‐binding proteins continues to expand, with the discovery of new cdG‐binding targets, such as the YajQ family protein XC_3703 in *Xcc* (An *et al*., 2014b) and the type III injectisome ATPase HrcN in *P. syringae* (Trampari *et al*., 2015).

Plant‐pathogenic bacteria display evolved cell–cell signalling systems with multiple, simultaneous and overlapping input signals, non‐linear gene regulation and extensive crosstalk between signalling systems and between different bacterial species on the plant surface. It is therefore very difficult to effectively define individual signalling pathways, or to assign discrete functions to these pathways during infection. Nonetheless, as our understanding of intracellular signalling increases and additional experimental and analytical tools become available, the critical role of bacterial signalling pathways to plant pathogenesis will undoubtedly become much clearer.

## Adapting to the Plant Environment

Downstream of the signalling pathways that integrate pathogen responses to the environment are transcriptional changes associated with life on plant surfaces. Marco *et al*. (2005) have shown that genes involved in virulence, membrane transport, nutrient acquisition and intracellular signalling are up‐regulated in *Pss* B728a on the surface of bean plants. A subsequent microarray analysis of *Pss* B728a isolated from different leaf sites (Yu *et al*., 2013) showed that epiphytic locations specifically favour flagellar motility and the production of surfactants to facilitate surface swarming, chemosensing and chemotaxis, suggesting active microbial relocation across the leaf surface. Increased expression of phenylalanine degradation loci was also observed, possibly as a countermeasure to phenylpropanoid‐based plant defences. In contrast, apoplastic growth was linked to the expression of γ‐aminobutyric acid (GABA) metabolic genes, antioxidant production and the biosynthesis of secondary metabolites, for example, the lipodepsipeptide phytotoxins and syringolin A (Yu *et al*., 2013).

A further study by Freeman *et al*. (2013) examined differences in transcriptional responses between two *P. syringae* strains, *Pss* B728a and *Pto* DC3000, on osmotic shock, and used these data to explain the observed differences in epiphytic survival between the two strains. Consistent with its higher survival rate on the leaf surface, *Pss* B728a exhibited increased osmotolerance relative to *Pto* DC3000, and higher rates of uptake of plant‐derived osmoprotectants. The two strains also displayed markedly distinct transcriptional responses to osmotic shock; multiple loci were up‐regulated in *Pss* B728a, including type VI secretion system (T6SS) and alginate biosynthesis pathways, whereas *Pto* DC3000 showed no change or repression of these loci and down‐regulated expression of T3SS genes (Freeman *et al*., 2013). Based on this, Freeman *et al*. (2013) suggested that epiphytic competence was mediated by strong, actively induced osmotolerance, accompanied by the synthesis of alginate and the T6SS.

A particularly well‐studied example of a plant environment‐sensing regulatory pathway is the induction of virulence gene expression in *Ag. tumefaciens* by low pH, plant‐associated sugars and phenolics, such as acetosyringone (Lee *et al*., 1995; Peng *et al*., 1998). Expression of the *vir* regulon is controlled by the two‐component VirAG regulatory system, consisting of a homodimeric transmembrane histidine protein kinase (VirA) which directly binds to acetosyringone and other target phenolic molecules (Lee *et al*., 1995), and controls the transcriptional regulator VirG (Wise and Binns, 2015). Sugar moieties are sensed by the accessory periplasmic glucose–galactose‐binding protein ChvE, which interacts directly with VirA and enhances its sensitivity to phenolics (Peng *et al*., 1998). The VirAG/ChvE pathway enables *Agrobacterium* to specifically induce virulence genes in response to specific chemical signals synthesized at plant wound sites, and so to effectively induce the formation of crown gall tumours.

The examination of individual pathogens in defined model plant systems has yielded an enormous amount of valuable information on the pathways that govern plant surface association and infection. However, these studies are, by necessity, highly simplified models for phytopathogen interactions with crops in the field. In a natural environment, pathogenic behaviour is extensively modulated by interactions with other constituents of the plant microbiome, with both synergistic and antagonistic interactions markedly affecting epiphytic survival and plant infection (Andrews and Harris, 2000; Delmotte *et al*., 2009; Ritpitakphong *et al*., 2016). An understanding of pathogen infection and plant surface colonization in the context of the whole microbiome represents a huge and challenging new frontier in plant pathogenicity research. To address this challenge, recent advances in sequencing and the development of additional, advanced techniques for the interrogation of bacterial populations are starting to be used to shed light on the distribution and behaviour of whole populations of bacteria, both pathogenic and commensal, on plant surfaces. Sequencing‐led techniques now enable us to analyse the composition of the leaf surface‐associated microbiome and to determine its impacts on pathogenicity (Ritpitakphong *et al*., 2016), whereas metatranscriptomic (Molina *et al*., 2012) and proteomic analysis (Delmotte *et al*., 2009) techniques may be used to examine the relevant bacterial systems for epiphytic life at the population level, and to map the regulatory transition from surface survival to migration into the apoplast. Finally, quantitative metabolomics techniques, such as imaging mass spectroscopy and quantitative nuclear magnetic resonance (Ryffel *et al*., 2015), have been used to analyse the *in situ* distribution of small molecules and metabolites in the leaf surface microbiome, and how these change on pathogen infection.

## Migrating Across Leaf Surfaces and into the Apoplast

Many pathogenic bacteria respond to leaf surface contact by expressing traits that promote survival until environmental conditions allow the penetration into the apoplast. Once such conditions arise, or immediately in the case of some poor epiphytes, pathogens deploy various motility systems to allow them to migrate across the leaf surface to stomata, wounds and other access sites to the interior of the plant. Flagella‐driven motility confers epiphytic fitness advantages in *P. syringae* (Haefele and Lindow, 1987), and is highly important for surface colonization and effective plant virulence (Tans‐Kersten *et al*., 2001). Flagella gene expression is typically coordinated with chemotaxis (Yu *et al*., 2013) and the production and deployment of surfactant molecules that enable the bacteria to swarm across solid surfaces, such as leaves (Burch *et al*., 2012). The importance of flagellar motility primarily during the early stages of infection is supported by infection data for both the soil‐borne pathogen *R. solanacearum* (Tans‐Kersten *et al*., 2001) and *P. syringae* (Pfeilmeier *et al*., 2016). In both studies, suppression of flagella biosynthesis, or deletion of flagella genes, led to significantly compromised plant infections, but only when bacteria were sprayed/soaked onto intact plants. When bacteria were directly applied to cut petioles (Tans‐Kersten *et al*., 2001), or when the requirement for apoplastic penetration was bypassed by syringe infiltration of bacteria (Pfeilmeier *et al*., 2016), no loss of virulence was seen in either case. Interestingly, the deletion of several signalling genes [*P. syringae rimK* (Little *et al*., 2016), *rpfS* from *Xcc* (An *et al*., 2014a) and *xbmR* from *X. citri* ssp. *citri* (Yaryura *et al*., 2015)] produces similar, conditional effects, with virulence defects only seen on bacterial application to leaf surfaces. In addition to flagella, type IV pili are required for bacterial biofilm formation and attachment, twitching motility and virulence in pathogens, including *P. syringae* (Nguyen *et al*., 2012) and *R. solanacearum* (Kang *et al*., 2002).

The importance of motility during plant infection is well established. However, it is equally important that flagella production is tightly controlled, both to enable the efficient and directional migration of bacteria into the apoplast and to avoid detection by plasma membrane‐localized pattern recognition receptors (PRRs), which initiate defence responses collectively referred to as PRR‐ or pattern‐triggered immunity (PTI) (Macho and Zipfel, 2014). For this reason, flagella are under tight regulatory control, at both the level of production and expression, and through the degradation of excess flagellin monomers by the alkaline protease AprA (Pel *et al*., 2014). The control of motility and the regulation of the expression of flagella/pili/surfactant loci at appropriate times during plant infection are increasingly being recognized as critical components of plant pathogenicity. How these processes function *in planta* is currently not fully understood, and is the focus of increasing investigation.

Alongside more conventional genetics‐led approaches, future research towards a full understanding of motility control and flagella gene expression during plant infection is likely to use increasingly sophisticated fluorescence microscopy and other visualization methods to examine pathogen spread *in planta*, together with emerging technologies that harness the increasing power and accessibility of next‐generation sequencing. An exciting new example of this is Sequence Tag‐based Analysis of Microbial Populations (STAMP) (Abel *et al*., 2015), which uses libraries of heterologously tagged microbes to quantify population bottlenecks and to infer founding population sizes for a given infection. Although STAMP has so far been mainly used for the analysis of intestinal infections with human pathogens, the technique could easily be adapted to examine the population dynamics of phytopathogen infection and dissemination in plant tissue. In principle, this technique could also be used to conduct simultaneous assays for competitive *in planta* infection by multiple pathogens/mutant strains, and has the potential to substantially increase the resolution and accuracy of research into the bacterial pathways that mediate plant infections.

## Getting Inside the Plant—Stomatal Hijacking and Cell Wall‐Damaging Enzymes

To initiate an infection, free‐living bacterial pathogens must overcome the plant's surface defences and gain entry into the apoplast. One obvious entry point for bacteria is the stomata. However, plants close their stomatal pores as part of their innate immune response, preventing bacterial ingress (Gudesblat *et al*., 2009a; Melotto *et al*., 2006). In response, many phytopathogens, for example *Xcc* (Gudesblat *et al*., 2009b) and numerous *P. syringae* pathovars (Melotto *et al*., 2006), produce and secrete phytotoxins that overcome stomatal immunity. A number of different pathogen‐secreted molecules act as antistomate defence factors, including the well‐studied toxins coronatine and syringolin A (Melotto and Kunkel, 2013; Zeng and He, 2010). In each case, these phytotoxins function by interfering with NPR1‐dependent salicylic acid (SA) signalling (Xin and He, 2013).

In addition to entry through the stomata, phytopathogens can enter the apoplast through wounds on the plant epidermis. To this end, many pathogens produce enzymes and other proteins that damage the cell wall and thus allow entry into the plant tissue. Several pathovars of *P. syringae* (Gaignard and Luisetti, 1993) produce ice nucleating agents (INAs), and *INA* loci have also been detected in other pathogenic species, such as *X. campestris* (Gurian‐Sherman and Lindow, 1993) and *Pantoea ananatis* (Sauer *et al*., 2014). The size of the protein, its membrane association and tendency to aggregate *in vitro* have so far confounded efforts to produce a protein structure for a bacterial INA (Garnham *et al*., 2011), and our understanding of their structure and function is largely based on *in silico* analysis. These models propose that INAs order water molecules into an ice‐like clathrin lattice that raises the temperature at which ice nucleates, causing water to freeze at high sub‐zero temperatures (Garnham *et al*., 2011). Frost damage caused by induced ice nucleation allows pathogen entry into plant tissue. Genes conferring freeze–thaw resistance have also been shown to be widespread among epiphytic bacteria (Wu *et al*., 2012), which would contribute to the survival of these microbes under both abiotic and pathogen‐induced frost conditions, and is consistent with the idea that directed frost damage is a common trait among phytopathogens.

In contrast with the epiphytic lifestyle of *Xanthomonas* and *Pseudomonas* spp. at the beginning of an infection, other bacterial phytopathogens, such as *Xy. fastidiosa* (Chatterjee *et al*., 2008a) and *Phytoplasma* (Sugio and Hogenhout, 2012), use sap‐feeding insects as vectors for the penetration of host tissue and as a dissemination mechanism. Phytopathogens also release numerous enzymes, mainly through type II secretion systems (Korotkov *et al*., 2012), which break down plant cell wall structural molecules and hydrolyse the connective lamellae between individual plant cells, providing a carbon source for the pathogen, as well as a further mechanism for apoplastic propagation and spread through the plant tissues. A good example of this latter function is the degradation of intervessel pit membranes in grapevine xylem by *Xy. fastidiosa*, allowing systemic expansion by the pathogen (Sun *et al*., 2011). Extracellular degradative enzymes are structurally and functionally diverse, and include proteases, cellulases, pectinases and xylanases (Dejean *et al*., 2013; Lee *et al*., 2014; Toth *et al*., 2003). These cell wall‐degrading enzymes are particularly associated with *Phytoplasma*, *Xylella* and *Xanthomonas* spp., and soft‐rot pathogens from the genera *Erwinia* and *Pectobacterium*. These bacteria all secrete complex mixtures of enzymes and contain multiple functionally redundant isoenzymes, which has so far presented difficulties in understanding the exact contribution of individual enzymes to pathogenesis (Toth *et al*., 2003).

## Plant Manipulation by Pathogenic Bacteria

In addition to degradative enzymes, phytopathogenic microbes produce small, secreted phytotoxins that contribute to bacterial virulence by subverting host defence responses and increasing chlorosis and tissue necrosis. Some phytotoxins cause direct damage to plant cells, whereas others modulate metabolic and signalling pathways in the host to benefit the invading pathogen. Lipodepsipeptide toxins, such as the syringomycins and syringopeptins, are examples of the former. They are produced by non‐ribosomal peptide synthetases during *P. syringae* plant infections, and are amphipathic in nature, inducing tissue necrosis by forming pores in the plasma membranes of plant cells (Melotto and Kunkel, 2013). The modified peptide toxins phaseolotoxin, mangotoxin and tabtoxin are produced by various *P. syringae* pathovars, and induce chlorosis and necrosis in plant tissue. These toxins target the enzymes involved in amino acid biosynthesis, inhibiting their activity and interfering with nitrogen metabolism. The resulting accumulation of nitrogen‐containing intermediates can be utilized by the pathogen as a food source (Arrebola *et al*., 2011). The toxic moiety of tabtoxin is released by hydrolysis once inside the plant cell, and induces the degradation of chlorophyll, leading to tissue yellowing and chlorosis through the irreversible inhibition of glutamine synthetase (Langston‐Unkefer *et al*., 1987). Phaseolotoxin functions via a similar mechanism, although, in this case, the toxic hydrolytic moiety, octicidine, inhibits ornithine carbamoyl transferase, leading to severe yellowing and tissue chlorosis as a result of metabolic unbalancing of the plant cell (Bender *et al*., 1999).

Pathogens interfere with hormone physiology in their hosts in order to maximize virulence and overcome plant defences, and manipulation of these internal signalling pathways is clearly central to effective pathogenesis. Many phytotoxic molecules produced by pathogenic microbes have both structural and functional parallels to auxin and other plant hormones. A good example of this is the polyketide plant hormone mimic coronatine, which, in addition to inducing stomatal opening, promotes apoplastic proliferation (Zeng and He, 2010) and enhances disease symptom development. Coronatine is recognized by the plant COI1/JAZ receptor complex and stimulates jasmonic acid (JA) signalling in the plant, in turn suppressing SA‐mediated defences (Xin and He, 2013). Many of the biological effects of coronatine closely resemble those induced by jasmonates. These include the stimulation of chlorosis, anthocyanin production, inhibition of root growth and induction of various JA‐responsive genes. Coronatine activity is also associated with several non‐JA/SA‐mediated virulence phenotypes. It contributes to chlorosis and yellowing through stimulation of the plant STAYGREEN gene (Mecey *et al*., 2011), as well as inducing necrotic cell death by increasing the production of reactive oxygen species (Ishiga *et al*., 2009).

Several plant pathogens also encode enzyme pathways that directly synthesize plant hormones, providing a direct mechanism for the manipulation of plant hormone signalling. The production of cytokinins, abscisic acid (ABA), indole acetic acid (auxin), JA and ethylene has been identified in various bacterial species (Robert‐Seilaniantz *et al*., 2011). There is a substantial and complex level of crosstalk between plant hormone signalling pathways, and phytopathogens are able to exploit this by producing or suppressing different hormones, thereby manipulating plant defences and metabolism to their advantage (Robert‐Seilaniantz *et al*., 2011). Operons for the biosynthesis and catabolism of auxin have been found in several phytopathogens, such as *Ag. tumefaciens*, *Pseudomonas savastanoi*, *P. syringae* and *Pantoea agglomerans* (Spaepen and Vanderleyden, 2011). Increased free auxin levels appear to benefit phytopathogens by suppressing host defence responses, and thus promoting pathogen susceptibility (Navarro *et al*., 2006; Spaepen and Vanderleyden, 2011). Another plant hormone produced by various phytopathogens is the stress response factor ethylene. Ethylene has been reported to both stimulate and suppress plant defence responses, depending on the particular interactions tested, although, for *P. syringae* and other virulent phytopathogens, ethylene production generally appears to promote disease (Melotto and Kunkel, 2013).

As the number of sequenced phytopathogen genomes continues to rapidly rise, we are likely to see the increasing importance of comparative genomic analysis tools (Martinez‐Garcia *et al*., 2015), alongside predictive algorithms for small‐molecule biosynthetic pathways and enzymes. Perhaps the most well established of these is AntiSMASH 3.0 (Weber *et al*., 2015), which identifies gene clusters and biosynthetic enzymes for natural products. *In silico* techniques are already extensively used in natural product operon discovery, and are likely to see increasing use in plant pathogen biology in years to come. Both the existence and, increasingly, the function of plant‐pathogenic molecules and peptides will become ever more amenable to *in silico* analysis, and this method is likely to become the primary approach by which future phytotoxin discoveries are made.

## Effector Proteins and Virulence

**Figure 4:**
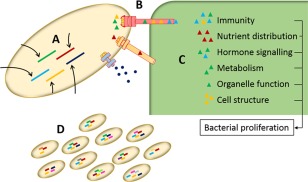
Schematic overview of important processes associated with bacterial effectors. (A) The regulation of virulence gene expression by extracellular and intracellular stimuli. (B) Coordinated translocation of effectors by secretion systems, such as the depicted type III secretion system (T3SS), T4SS and T2SS. (C) Effectors target various host processes to promote bacterial proliferation. These processes include immunity, nutrient distribution, hormone signalling, plant metabolism, organelle function and cell structure. (D) Host–pathogen interactions drive evolutionary mechanisms, leading to the expansion and diversification of virulence gene families and shaping the effector repertoire of pathogen populations.

Successful infection by many phytopathogenic bacteria depends on the secretion of diverse virulence factors, including enzymes, toxins and other host‐manipulating molecules, into the extracellular milieu or directly into the host cytosol. Hemibiotrophic bacteria, such as *Xanthomonas* spp. and *P. syringae*, use a combination of different secretion systems to enable the export and delivery of effectors, which are secreted proteins that manipulate the structure or function of host components, to the appropriate location for a coordinated and effective infection (Chang *et al*., 2014). In the next section, we discuss important questions relating to bacterial effectors, their translocation and ongoing attempts to understand the complex molecular interaction between plants and bacterial pathogens (Fig. 4).

## Regulation, Dynamics and Hierarchy of Effector Translocation

Coordination between available secretion systems contributes markedly to phytopathogen versatility. However, we currently have only a limited knowledge of how the different secretion systems in a bacterial cell are regulated in response to changes in external stimuli (Fig. 4A), with a few exceptions. In *P. aeruginosa*, the post‐transcriptional regulator RsmA acts as a molecular switch between acute and chronic infections by negatively affecting the translation of *pel*, *psl* and T6SS mRNAs and by promoting flagellar, T4SS and T3SS gene transcription (Brencic and Lory, 2009; Moscoso *et al*., 2011). Similarly, the sensor kinases RetS and LadS antagonistically regulate T6SS and T3SS in *Pss* B728a (Records and Gross, 2010). QS plays a critical role in the regulation of T2SS in *Xanthomonas* (Jha *et al*., 2005). Regulation of the so‐called *hrp* (*hypersensitive response and pathogenicity*) regulon, which includes T3SS structural genes, T3SS‐associated regulators and many T3Es, has also been studied extensively (Büttner and Bonas, 2010; Genin and Denny, 2012; Tampakaki *et al*., 2010).

In addition to the transcriptional regulation of T3SS, pathogens ensure hierarchical and temporal control of T3E delivery during infection by post‐translational mechanisms (Galán *et al*., 2014; Lohou *et al*., 2013). *Salmonella enterica* uses a cytoplasmic sorting platform involving chaperones to recruit and secrete T3Es in a well‐defined order (Lara‐Tejero *et al*., 2011). Bacterial plant pathogens also employ *hrp‐associated* (*hpa*) proteins, such as chaperones and export control proteins, for coordinated delivery of T3Es into host cells (Lohou *et al*., 2013). Recently, a proteomics approach to the *R. solanacearum* secretome identified differential T3E secretion patterns in *hpaB*, *hpaD* and *hpaG* mutant strains, which affected bacterial virulence and supports the idea of the hierarchical delivery of T3Es (Lonjon *et al*., 2016). Mechanistically, it has been suggested that the global T3E export chaperone HpaB interacts with HpaA to select and guide T3Es to the T3SS‐associated ATPase HrcN for translocation (Büttner *et al*., 2006; Chang *et al*., 2014). Interestingly, the secondary messenger cdG has recently been shown to allosterically regulate HrcN activity, suggesting a role for this signalling molecule in the control of T3E translocation dynamics (Trampari *et al*., 2015). Coordinated secretion of T3Es is important for *Pss* B728a during epiphytic growth as, in this phase, HopAA1 and HopZ3 seem to play a special role in the colonization of distinctive leaf surfaces (Lee *et al*., 2012). To gain a detailed insight into the temporal dynamics of T3E secretion, a microscopy‐based assay tracking translocation of individual T3Es has been developed for *Escherichia coli* (Mills *et al*., 2013). This technique could be readily adapted for plant pathogens, and could potentially shed further light on exactly how these bacteria deploy their T3Es during the infection process.

## Different Modes of Effector Translocation

Coordinated translocation of proteins and other cargo molecules requires complex transport systems (Fig. 4B). There are at least six secretion systems employed by Gram‐negative bacteria to deliver proteins into the extracellular environment or directly into a recipient cell. These secretion pathways, type I to type VI, differ significantly in their composition, recognition of cargo and function (Büttner and Bonas, 2010; Chang *et al*., 2014). T3SS, in particular, plays an essential role in pathogenicity, as mutants in many Gram‐negative phytopathogenic species fail to cause disease in susceptible plants. Given the importance of T3SS during pathogenesis, specific attention has been dedicated to identify T3E repertoires and their biological function. However, other secretion systems have been shown to be relevant and, in some cases, crucial for pathogenesis. Often, multiple secretion systems work alongside each another to achieve full virulence (Preston *et al*., 2005). Future research will reveal the effector repertoires and *in planta* regulation of the complete phytopathogenic secretome, contributing to a better understanding of bacterial pathogenesis.

The advent of whole‐genome sequencing has made it possible to identify an organism's complete T3E repertoire by making use of conserved elements and characteristic signatures in the genome (Lindeberg *et al*., 2006). Over recent years, various genome mining approaches, coupled with functional characterization, have allowed us to build up a provisional T3E super‐repertoire for *P. syringae* (O'Brien *et al*., 2011), *Xanthomonas* spp. (White *et al*., 2009) and *R. solanacearum* (Peeters *et al*., 2013; Roux *et al*., 2015). Although the majority of T3E families from the *P. syringae* species complex have been described (Baltrus *et al*., 2011), new T3E families are continuously being found (Hockett *et al*., 2014; Matas *et al*., 2013), with low‐cost draft genomes enabled by next‐generation sequencing likely to permit more extensive sampling from different crop species, alternative hosts or environmental samples in the future. Community efforts have established a unified nomenclature, built up databases of known T3E families and generated bioinformatics tools for extensive genomic analysis (Lindeberg *et al*., 2005; Peeters *et al*., 2013), which should greatly assist future efforts to identify and characterize effector proteins.

Pathogenic and non‐pathogenic bacteria use T2SS to deliver proteins into the extracellular environment (Johnson *et al*., 2006). A large number of T2Es are major virulence factors, such as toxins, proteases and cell wall‐degrading enzymes, contributing to virulence in animals and plants. Plant pathogens containing a T2SS include numerous members of the genera *Erwinia*, *Pseudomonas*, *Ralstonia* and *Xanthomonas* (Cianciotto, 2005; Jha *et al*., 2005). All sequenced *Xanthomonas* species and many other Gram‐negative bacteria contain the *lipA* gene encoding a type II secreted lipase, whose function has been shown to be required for the full virulence of *X. oryzae* pv. *oryzae* (Aparna *et al*., 2009) and *X. campestris* pv. *vesicatoria* (Tamir‐Ariel *et al*., 2012).

T4SSs have been found in diverse bacterial genera, and translocate DNA and proteins into the extracellular environment or directly into recipient cells (Bhatty *et al*., 2013). T4SS cargo can include proteins, DNA–protein complexes and fully assembled protein macro‐complexes (Bhatty *et al*., 2013). Among plant pathogens, T4SS is mostly known in *Ag. tumefaciens* for translocation of T‐DNA into host cells to enable tumour induction (Gohlke and Deeken, 2014). Recently, it has been shown that *X. citri* uses a T4SS for toxin translocation to kill other bacteria, providing a growth advantage in mixed communities (Souza *et al*., 2015). Genome analysis has also identified putative T1SS, T5SS and T6SS in diverse plant pathogens; however, their biological roles are currently poorly understood, with no strong virulence phenotype associated with any of these secretion systems so far (Chang *et al*., 2014; Preston *et al*., 2005; Records and Gross, 2010). Future genome comparison analyses and refined bioinformatics approaches will tell us more about the variation within the different phytopathogen secretory pathways and their associated effectors, and will hopefully reveal their roles during pathogenesis, and also during environmental persistence (Bhatty *et al*., 2013; Korotkov *et al*., 2012; Sarris *et al*., 2010). Research on non‐culturable phytopathogens, such as the obligate intracellular *Phytoplasma*, have particularly benefited from genome mining approaches. This has enabled the discovery of many secreted effectors with crucial functions for the pathogen lifecycle (MacLean *et al*., 2011; Sugio and Hogenhout, 2012).

## Function of T3ES

Effectors interfere with plant processes to benefit microbial fitness in the host environment. Genome analysis has revealed whole suites of effector proteins, whereas functional characterization has revealed that a major role of T3Es is the suppression of plant immune responses (Block and Alfano, 2011; Cunnac *et al*., 2009; Macho and Zipfel, 2015).The molecular interplay between bacterial T3Es and plant immune components has been summarized in a model describing the evolutionary arms race of pathogens and plants (Dodds and Rathjen, 2010; Jones and Dangl, 2006). Although T3Es suppressing plant immunity have been studied in great detail, other host processes that are frequently targeted by effectors during the course of an infection, but are not directly linked to immunity, have been less well characterized. These process include nutrient distribution, metabolism, organelle function and cell development (Fig. 4C) (Macho, 2016). In addition to classifying effectors by their degree of conservation, assigning them to effector‐targeted pathways (ETPs) according to their function might provide better insights into the complex processes and determinants underlying virulence, as well as host and tissue specificity (Win *et al*., 2012).

The synchronization of host physiological status and bacterial metabolic pathways is necessary for optimal pathogen growth. To this end, transcription activator‐like (TAL) effectors are encoded by members of *Xanthomonas* and *Ralstonia* spp., and function as plant transcription factors to promote virulence and to establish a compatible environment to support bacterial growth (Bogdanove *et al*., 2010). For example, the rice pathogen *X. oryzae* pv. *oryzae* manipulates the expression genes encoding for SWEET sugar transporters by multiple TAL effectors (Chen *et al*., 2010; Streubel *et al*., 2013). Interestingly, *Pto* DC3000 also induces T3SS‐dependent expression of the *SWEET* genes during infection of *Arabidopsis thaliana* (Chen *et al*., 2010). The induced sugar efflux from plant cells to the apoplast might also result in water release to maintain osmotic homeostasis in the tissue, further changing the environment in the intercellular space and specifically benefiting bacterial infection (Macho, 2016). Pathogens can also modify plant tissue by manipulating developmental regulators or plant hormone signalling. For example, the TAL effector AvrBs3 from *X. campestris* induces expression of the plant cell size regulator *UPA20*, resulting in enlarged mesophyll cells, which may promote bacterial growth and dissemination (Kay *et al*., 2007). Various T3Es from *P. syringae*, such as AvrRpt2, HopX1 and AvrPtoB, affect the auxin, JA and ABA signalling pathways, respectively (Cui *et al*., 2013; De Torres Zabala *et al*., 2009; Gimenez‐Ibanez *et al*., 2014). Interference with hormone signalling by deployment of T3Es is widespread among phytopathogens and may directly or indirectly disrupt immune responses, but also impacts plant physiology and metabolism, thereby creating a niche for bacterial propagation (Kazan and Lyons, 2014). Additional ETPs are the plant cytoskeleton or cellular trafficking pathways, with effector deployment leading to altered cell structure and function. These phenotypes have been associated with suppression of defence responses (Kang *et al*., 2014; Nomura *et al*., 2011); however, the remodelling of cell integrity and transport pathways can also have broader implications for plant homeostasis. T3Es have also been shown to directly affect plant metabolism by interfering with biosynthetic pathways for secondary metabolites. For example, *Pantoea stewartii* secretes a T3E of the AvrE family, which perturbs phenylpropanoid metabolism and promotes pathogen virulence (Asselin *et al*., 2015). Chloroplasts and mitochondria are involved in many aspects of plant physiology and are consequently an obvious target for T3Es. Among others, HopK1 from *P. syringae* localizes to the chloroplast, suppresses photosynthesis and contributes to virulence (Li *et al*., 2014). Similarly, *P. syringae* HopG1 perturbs mitochondrial function and plant development and probably contributes to bacterial virulence (Block *et al*., 2010). These examples highlight the various ways in which pathogens use T3Es to manipulate their hosts and to generate a suitable environment for survival, growth and dispersal. Finally, the manipulation of plant development represents an effective virulence strategy. *Agrobacterium tumefaciens* uses a T4SS to transfer DNA (T‐DNA) together with virulence proteins into plant cells. The T‐DNA integrates into the genome and governs host cell reprogramming, leading to altered metabolism and morphology (Gohlke and Deeken, 2014).

Typically, the T3E repertoire is functionally redundant and deletion of individual T3Es often has no effect on virulence. Systematic mutagenesis, combined with virulence phenotyping, has been used to dissect the function and importance of individual T3Es in *Pto* DC3000, leading to the identification of a minimal T3E repertoire, which is sufficient for wild‐type‐level infection of *Nicotiana benthamiana* (Cunnac *et al*., 2011; Kvitko *et al*., 2009). A major future challenge will be to elucidate what is required in a functional T3E repertoire to enable pathogenicity (Lindeberg *et al*., 2012). To address this, genomics and computational analysis must be coupled to functional and mechanistic studies to characterize the role of T3Es in the plant cell. Improved biochemical, proteomics and cell biology techniques, together with high‐throughput methods, such as transient assays in heterologous systems to screen for disease symptoms (Almeida *et al*., 2009; Cunnac *et al*., 2011), or to determine subcellular localization and host interactors (Petre *et al*., 2015), will also help us to understand the function and evolution of T3E repertoires.

## Evolution of Effector Repertoires

Phytopathogens, such as *P. syringae* or the *Xanthomonas* genus, have a broad host range, but pathogen variants (pathovars) or single species are restricted to one or a few plants, reflecting strong host adaptation (Lindeberg *et al*., 2009; Ryan *et al*., 2011). Understanding the factors that influence the host ranges of pathogenic bacteria and the underlying mechanism of host range evolution is a major challenge (Lindeberg *et al*., 2012).

Genome comparison between phylogenetically related strains provides insights into the evolutionary processes shaping the T3E repertoire, and its contribution to host and/or tissue specificity for individual strains. In this approach, conserved T3Es across phylogenetically diverse strains represent core T3Es that provide basal virulence functions in all hosts, whereas variable T3Es are important for individual host specificity (Lindeberg *et al*., 2012). A comparison between 19 *P. syringae* strains representing the three major subclades revealed a core set of the four bona fide T3Es: HopM, AvrE, HopAA and HopI (Baltrus *et al*., 2011). However, the authors could not pinpoint factors determining host specialization because of the high diversity of virulence components and the variability of T3E repertoires among different pathovars (Baltrus *et al*., 2011).

The molecular arms race between pathogens and hosts drives the evolution of T3Es as immune suppressors (Guo *et al*., 2009; Lindeberg *et al*., 2012; Win *et al*., 2012). However, the evolutionary purpose of the high degree of functional redundancy observed in T3E repertoires is not fully understood. Targeting a plant defence pathway multiple times might ensure the effective disruption of plant defences over the course of an infection and would provide a robust long‐term virulence strategy in case a specific T3E is recognized by a host species and turned into an avirulence effector. A model for two‐stage co‐evolution has been suggested, in which variable T3Es evolve to protect the function of core T3Es, leading to highly redundant repertoires (Lindeberg *et al*., 2012). Furthermore, a pathogen population benefits from heterogeneous, strain‐specific T3E repertoires when disease‐resistant host plants emerge. Under these conditions, individual strains with virulent T3Es for the new host are positively selected and help to ensure the survival of the pathogen population. This evolutionary ‘bet hedging’ strategy would allow a population to include redundant T3Es to increase flexibility in response to evolving host plants (Win *et al*., 2012).

No clear pattern was detected in the T3E repertoire that could be associated with the specificity of *P. syringae* pathovars (Lindeberg *et al*., 2012), *R. solanacearum* strains (Genin and Denny, 2012) or *Xanthomonas* spp. (Roux *et al*., 2015), underlining the complex genetic basis for adaptation to specific hosts. This is also exemplified by the diverse T3E repertoires from individual isolates. The closely related *Pto* strains DC3000 and T1 can both infect tomato plants with a shared T3E repertoire of 14 T3E genes, in contrast with 15 and 11 additional strain‐specific T3E genes, respectively (Almeida *et al*., 2009). To address the genetic basis of why *Pto* DC3000, but not T1, is able to infect *A. thaliana*, mutagenesis and functional T3E characterization were performed, but could not fully explain the compatibility of *Pto* DC3000 with *A. thaliana*. Additional factors might be involved in pathogenesis on *A. thaliana* (Almeida *et al*., 2009; Sohn *et al*., 2012). Interestingly, *P. syringae* phylogroup II strains contain the smallest number of T3Es, but encode operons for many phytotoxins compared with the other groups (Baltrus *et al*., 2011).

Diversification of the T3E repertoire is an important long‐term virulence strategy, and pathogenic bacteria have evolved several mechanisms for the acquisition and loss of T3E genes (Fig. 4D) (Jackson *et al*., 2011); therefore, an analysis based on only the presence or absence of T3E genes might not provide the full picture. Several examples have been reported in which pathogenic bacteria modify T3E gene expression, protein activity, target specificity or detection by *R* proteins (Ma and Guttman, 2008). Comparative genomic analysis complements research into molecular plant–microbe interactions, as it can provide additional information about discrete residues in a given T3E under diversifying selection imposed by the host plant (Baltrus *et al*., 2011).

A robust association between host range evolution and T3E repertoire requires many genomic sequences and careful sampling. A population genomics approach, in which isolates ideally represent a defined population, helps us to understand how host shifts and specialization occur and what evolutionary paths are available when a pathogen adapts to a specific host (Vinatzer *et al*., 2014). Ultimately, examination of the evolution of T3Es provides a very detailed picture of the adaptation process, as T3Es are highly variable and evolve rapidly because of strong selection pressure imposed by plant immunity components (Ma and Guttman, 2008). Progress in bioinformatics methods for T3E detection, together with functional information on T3Es and population genomics analysis determining phylogenetic relationships between isolates, will enable us to monitor pathogen populations in the field and to predict epidemic outbreaks in the future (Vinatzer *et al*., 2014).

## Conclusion

Although the future challenges faced by plant pathology researchers are certainly daunting, new research tools, including techniques facilitated by the rise of next‐generation sequencing and advanced computational analysis, have already enabled us to investigate plant pathogenesis at a resolution that would have been impossible just a few years ago. Future research is likely to continue to move towards a global understanding of the pathogen–plant interaction, and promises to keep us busy for many years to come.
